# Modelling Climate Change Impacts on the Seasonality of Water Resources in the Upper Ca River Watershed in Southeast Asia

**DOI:** 10.1155/2014/279135

**Published:** 2014-08-17

**Authors:** Pham Quy Giang, Kosuke Toshiki, Masahiro Sakata, Shoichi Kunikane, Tran Quoc Vinh

**Affiliations:** ^1^Department of Environmental and Life Sciences, University of Shizuoka, 52-1 Yada, Suruga-ku, Shizuoka-shi, Shizuoka 422-8526, Japan; ^2^Faculty of Land Management, Vietnam National University of Agriculture, Hanoi 131000, Vietnam

## Abstract

The impact of climate change on the seasonality of water resources in the Upper Ca River Watershed in mainland Southeast Asia was assessed using downscaled global climate models coupled with the SWAT model. The results indicated that temperature and evapotranspiration will increase in all months of future years. The area could warm as much as 3.4^°^C in the 2090s, with an increase of annual evapotranspiration of up to 23% in the same period. We found an increase in the seasonality of precipitation (both an increase in the wet season and a decrease in the dry season). The greatest monthly increase of up to 29% and the greatest monthly decrease of up to 30% are expected in the 2090s. As a result, decreases in dry season discharge and increases in wet season discharge are expected, with a span of ±25% for the highest monthly changes in the 2090s. This is expected to exacerbate the problem of seasonally uneven distribution of water resources: a large volume of water in the wet season and a scarcity of water in the dry season, a pattern that indicates the possibility of more frequent floods in the wet season and droughts in the dry season.

## 1. Introduction

In recent years, the consensus of natural scientists on the human-induced nature of climate change has become stronger as more evidence on the issue has accumulated. The Intergovernmental Panel on Climate Change (IPCC) has reported with what they describe as “virtual certainty” (probability >99%) that the Earth's mean surface temperature has increased by 0.4 to 0.8°C since the Second Industrial Revolution began around 1860 [1]. Most of this warming has occurred in two periods: from 1910 to 1945 (0.14°C/decade) and since 1976 (0.17°C/decade) [2]. Globally, nine of the ten warmest years since the Second Industrial Revolution have occurred since 1990 [3]. The IPCC also reported, with a probability of 90–99%, that there was an increase in precipitation in the mid-to-high latitudes of the Northern Hemisphere in the last century [1]. The primary cause of climate change is attributed to the emission of greenhouse gases (GHGs) due to the burning of fossil fuels, leading to an increase in the so-called greenhouse effect that occurs as a consequence of the unbalanced presence of GHGs in the atmosphere [4]. In addition, increases in aerosol concentration in the troposphere due to increases in the emission of gases such as sulphur and nitrogen oxides, as well as smoke and soot from fossil fuel burning, have also been found to impact the climate system, although the impact is regional and seasonal, associated with emission source and aerosol residence time in the atmosphere [5]. In general, since aerosols reflect and absorb incoming solar radiation, they drive a cooling effect at the Earth's surface and a warming effect in the troposphere [6]. Rosenfeld et al. found that aerosols caused a delay of early rainfall through their effect on clouds, resulting in greater amounts of cloud water and rain intensities later [7]. As the global emission of GHGs, as well as other air pollutants, is increasing due to population growth, urban expansion, industrial development, and so forth, it is predicted that global climate change is likely to become increasingly severe. Temperature is expected to increase by 1.8 to 4.0°C by the end of this century [8].

Water is widely acknowledged as one of the first factors to be affected by climate change [9]. Globally, there is growing evidence that the water cycle is being significantly altered [10]. First, precipitation is generally expected to increase worldwide, especially at higher latitudes [1]. Global warming is also predicted to intensify potential evapotranspiration (PET). Budyko estimated that PET would increase by 4 percent for every degree Celsius increase in temperature [11]. As the climate warms, snow cover is projected to contract and decrease, and glaciers and ice caps are projected to lose mass [9]. Based on simulations of 11 glaciers in various regions of the world, Schneeberger et al. projected a loss of 60% in the volume of these glaciers by 2050 [12]. Other impacts of a warmer climate on the water cycle include reduction of soil moisture, changes in the regime of groundwater discharge and recharge, and change in river runoff [9]. Thus, climate change impacts water cycle through various factors, and analysis of these impacts is a wide issue. A wide variety of studies on hydrological changes due to climate change have been published for numerous regions around the world, but not many of those studies have focused on the seasonal trends of river flow regimes, especially for transnational watersheds. In addition, previous studies have mostly focused on the impact of climate change on river discharge in which glacier-melt water contributes a substantial part [13–17], while few have focused on nonglacier dominated catchments. These reflect a gap in research. Furthermore, although climate change is a global issue, its impact varies from region to region and from country to country. Countries in climate-sensitive regions present higher vulnerability. In recent decades, most Asian countries have experienced more frequent extreme-weather events, including floods and droughts as a consequence of climate change and anthropogenic activities. This situation is projected to increase in the future [18–22]. Due to its geographic location, Vietnam is projected to be among countries most threatened by climate change impacts [23].

In terms of water resources, Vietnam possesses a dense river network, with 2,372 rivers that are longer than 10 km, comprising 13 large river systems. However, this river network includes many international rivers that originate in other countries, with only 28 percent of the total catchment area and 40 percent of the total annual volume of water flow belonging to Vietnam [24]. Furthermore, despite the high total annual runoff of over 800 billion m^3^, water shortages are common in many areas due to uneven regional and seasonal distributions of water resources. More than 60% of river water is concentrated in the Mekong River Delta, which covers only 12% of the total area of Vietnam, while the remaining less than 40% is spread over an area containing nearly 80% of the nation's population and over 90% of its production, trade, and service activities [24]. Seasonally, the average volume of rainfall in the wet season accounts for 75–85% of the total volume, while the dry season receives only the remaining 15–25% [25–27]. Future climate change is expected to exacerbate these conditions [24, 28].

Thus, the main objective of our study is to investigate if future climate change will intensify the seasonally uneven distribution of water resources in a transnational watershed, where climate change impact analysis is of importance not only for domestic water management but also for international cooperation. The upper part of the Ca River Watershed, which is shared between upstream Laos and downstream Vietnam, has been selected for our study. The Ca River Watershed is one of the nine main river systems of Vietnam and is the largest system in the North Central region of the country. However, it is important to note that the watershed is a typical example in terms of having a seasonally unbalanced distribution of water resources, as there are frequent floods in the wet season and droughts in the dry season. To achieve the study objectives, we employed a simulation approach, consisting of the Soil and Water Assessment Tool (SWAT) coupled with downscaled global climate model (GCM). Climate change projections for three time periods 2030s, 2060s, and 2090s, respectively, which represent near, middle, and far future, were generated under the IPCC emission scenarios B1, B2, and A2, using 20 GCMs integrated in the Model for the Assessment of Greenhouse-Gas Induced Climate Change/Regional Climate Scenario Generator (MAGICC/SCENGEN). A statistical downscaling method and the Monthly to Daily Weather Convertor (MODAWEC) were used to downscale the projected large-scale monthly climate data to local daily data which were then used for hydrological simulation. Hydrological simulation by SWAT was performed to assess future trends of river discharge, which would indicate the risk of flooding and drought to the watershed. The results of this study are expected to be useful for the development of effective water resources management strategies, especially for initiatives aimed at preventing the effects of flood and drought.

## 2. Materials and Methods

### 2.1. Study Watershed

**Figure 1:**
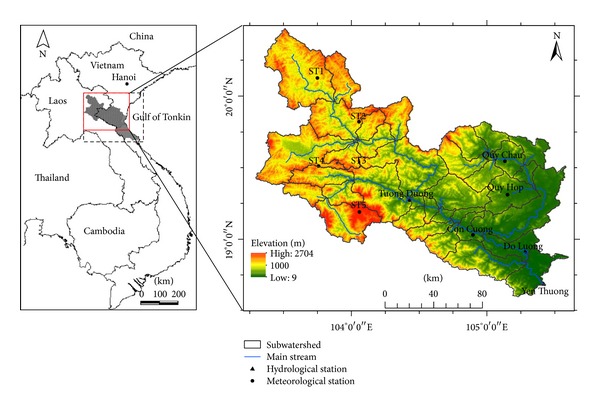
Geographic location of the entire Ca River Watershed (left) and the UCRW (right).

The Ca River originates in the Loi Mountains of Laos, crosses Laos PDR's Xiangkhouang Province, flows into Vietnam and through the province of Nghe An, and joins the La River before emptying into the Gulf of Tonkin at the Cua Hoi estuary. The river flows in a northwest to southeast direction and has a total length of more than 350 km, of which approximately 100 km is within the territory of Laos and 250 km is within Vietnam. The upper part of the Ca River Watershed (hereafter, the Upper Ca River Watershed—UCRW) defined in this study is the area of the watershed which has its outlet at Yen Thuong hydrological station (105°23′E, 18°41′N) in Nghe An Province (Vietnam) and covers an area of approximately 22,800 km^2^ of a total watershed size of 27,200 km^2^. Geographic location and detailed information about the UCRW are shown in Figure 1.

The UCRW is located in a tropical monsoon zone characterized by two distinct seasons. The wet season, starting from May to October, is hot and humid due to the southwest monsoon (locally called the Laos wind). Mean average temperature in this season is 27°C, with the highest temperature reaching 42°C around May to June. The dry season lasts the other six months, from November to April, and is cold and dry caused by the northeast monsoon. Mean average temperature in this season is 20°C, with the lowest temperature dropping to 2°C around January. According to the 40-year observation data from 1971 to 2010, mean annual precipitation in the UCRW is approximately 1,600 mm, of which the wet season accounts for more than 80%. Meanwhile, mean annual evaporation is 840 mm, with a mean humidity of around 80–85% (Table 2).

The area is dominated by rugged terrain with mountains on both sides of the main river reaching heights of around 10 m to 2,700 m above sea level. Soil types include mostly Humic Acrisols and Plinthic Ferralsols (accounting for around 80% of the total area), Rhodic Ferralsols, Lithic Leptosols, and Eutric Fluvisols. Except the alluvial soils in the low valleys, soils in the area are generally acidic, poor in nutrients, and highly susceptible to erosion [29]. The natural land cover is mostly evergreen and semideciduous tropical moist forest, although mixed forest can be found in some areas. Currently, around 40% of the UCRW remains forested. However, due to human disturbance, especially from logging activities and traditional shifting cultivation, this forest area is subject to decrease. The area's anthropogenic land cover includes annual rainfed crops (e.g., corn, groundnuts, and upland rice), irrigated rice, perennial crops (e.g., banana, sugarcane), orchards, pastures, bare soil, and residential areas.

The hydrology of the UCRW is typical of a tropical monsoon climate, with a high annual stream flow volume due to high annual rainfall, but there is highly uneven distribution through the year. The average annual flow volume measured at Yen Thuong Station is more than 17 billion m^3^, of which the wet season accounts for approximately 13 billion m^3^ (76%). Floods usually occur in the 3 last months of the wet season, August, September, and October, and very few cases have been recorded for November. The total volume of flow in these three months accounts for about 55% of the annual flow. In contrast, the months with the lowest flow are usually the last three months of the dry season, February, March, and April, with a total volume accounting for only about 7% of the annual flow. During the 40-year observation period (1971–2010) at Yen Thuong Station, the highest flow discharge was 9,140 m^3^/s and the lowest flow discharge was 2.67 m^3^/s.

### 2.2. Generation and Downscaling of Climate Change Scenarios

In this study, three emission scenarios from the IPCC Fourth Assessment Report (AR4) were used: A2, B2, and B1, respectively, representing high, medium, and low greenhouse gas emission levels [8]. Scenario A2 assumes a very heterogeneous future world, with continuously increasing population and regionally oriented economic development. Scenario B2 is also of a world with continuously increasing population, but at a rate lower than A2, and with intermediate levels of economic development. The emphasis of this scenario is on local rather than global solutions to economic, social, and environmental sustainability. Scenario B1 describes a world in which global solutions to economic, social, and environmental sustainability have been adopted. In this scenario, population reaches the peak in 2050 and declines thereafter. The use of the three scenarios A2, B2, and B1 in Vietnam was introduced by MONRE [30].

Future climate projection under the above three scenarios was generated using MAGICC/SCENGEN model version 5.3 [31]. MAGICC/SCENGEN is a coupled gas-cycle/climate model (MAGICC) that drives a spatial climate change scenario generator (SCENGEN). MAGICC is one of the primary models that have been used by the IPCC since 1990 to produce projections of future global-mean temperature and sea level rise. The climate model used in MAGICC is an upwelling-diffusion, energy-balance model that produces global and hemispheric-mean temperature output together with results for oceanic thermal expansion. Global-mean temperatures from MAGICC are then used to drive SCENGEN. SCENGEN uses a version of the pattern scaling method described by Santer et al. [32] to produce spatial patterns of change from a database of atmosphere/ocean GCM (AOGCM) data from the CMIP3/AR4 archive.

Out of 24 models of CMIP3, 20 GCMs are available in MAGICC/SCENGEN, and all of these 20 models were used in our study.  Table 1 shows the 20 models used. These models span latitude/longitude grid spacing in the range of about 1° to 4°, but in SCENGEN all of them were regridded to a common 2.5° by 2.5°. The result from SCENGEN displays changes in precipitation, temperature, and so forth, on each prediction grid cell. An observation dataset of surface temperature and precipitation was then used to validate model performance and calculate model weight.

Because the resultant data generated from MAGICC/SCENGEN have a coarse spatial resolution (2.5°  × 2.5°) and a monthly basis, downscaling methods were used to downscale these data to at-site daily data. First, a statistical downscaling method with conversion functions was used to transfer the large-scale monthly climate data to site-scale monthly data at local stations. In this study, the data was primarily downscaled to 5 local stations (as shown in Table 2), and downscaled data from these 5 stations was used to estimate data for other stations (i.e., ST1–ST5 shown in Figure 1) by the inverse distance weighting (IDW) interpolation method. The transfer function is a linear regression equation *y* = *ax* + *b*, where *y* is monthly temperature/precipitation observed at a local station, *x* is predicted monthly temperature/precipitation in the grid cell at the coordinates of the local station, and *a* and *b* are constants. The statistical downscaling for climate stations in Vietnam can be referred to in IMHEN [33] and MONRE [30].

Next, the downscaled monthly data at the local stations were downscaled again to daily data using the MODAWEC weather generator model [34]. In this model, to generate daily precipitation, a first-order Markov chain [35] is used to define the day as wet or dry. In the case of a wet day, daily precipitation is generated from a modified exponential equation. For temperature, the model developed by Richardson [36] is used to give first approximations of daily temperature because it simulates temperature that is correlated with precipitation. The residuals of temperature are generated from a multivariate normal distribution. Final values are then obtained by correcting the initial estimates using the average daily maximum and minimum temperature in a month.

The daily data for precipitation and temperature at local stations were then employed for hydrological simulation using the SWAT model. In this study, the baseline period was 1980–1999.

### 2.3. Hydrological Simulation and Data

The SWAT model is a physically based, semidistributed hydrological model based on a daily or a subdaily time step [37]. SWAT was developed for continuous simulation, as opposed to single event simulation models. The main purpose of SWAT is the computation of runoff and sediment and agricultural chemical yields in large complex watersheds with varying soils and land use management conditions over long time periods [37–39]. In this model, a watershed is divided into multiple subwatersheds that are then further subdivided into unique soil/land use characteristics called hydrologic response units (HRUs). SWAT requires a very large amount of data, including weather variables, topography, soil properties, land covers, and land management practices occurring in the watershed. However, SWAT has a weather generator module that is capable of generating daily data for precipitation, temperature, relative humidity, solar radiation, and wind speed from average monthly variables of these data. The module is therefore very useful for filling in missing daily data in the observed records.

In this study, weather data on a daily basis, including precipitation, temperature, solar radiation, and relative humidity, were available for five stations, that is, Con Cuong, Do Luong, Quy Chau, Quy Hop, and Tuong Duong. These data were collected from the Vietnam Institute of Meteorology, Hydrology and Environment (http://www.imh.ac.vn/) and the Hydro-Meteorological Data Center (http://www.hymetdata.gov.vn/). In addition, weather data at five points ST1–ST5 were obtained from the National Centers for Environmental Prediction (NCEP) Climate Forecast System Reanalysis (CFSR) (http://rda.ucar.edu/pub/cfsr.html/). Potential evapotranspiration (PET) was computed using the Hargreaves equation [40]. A digital elevation model (DEM) with a 3-arc-second resolution covering the entire Ca River Watershed was downloaded from the NASA Shuttle Radar Topographic Mission (http://srtm.csi.cgiar.org/). Land use data for the area inside Vietnam were collected from the General Department of Land Administration (http://www.gdla.gov.vn/) and the Provincial Department of Natural Resources and Environment of Nghe An Province (http://nghean.gov.vn/wps/portal/sotnmt). Land use data for the external part of the watershed in Laos were extracted from Landsat 7 image with a resolution of 30 m × 30 m obtained from the Global Land Cover Facility (http://glcf.umd.edu/data/landsat/). The land use data were then reclassified in accordance with the SWAT model input requirement. Soil data for the area in Laos were collected from the Harmonized World Soil Database (http://www.iiasa.ac.at/Research/LUC/External-World-soil-database/HTML/). Soil data for the area inside Vietnam were collected from the Soil and Fertilizers Research Institute (http://www.sfri.org.vn/Eng_Index.aspx) and Vietnam National University of Agriculture (http://www.vnua.edu.vn/eng/). The Vietnamese soil types were converted to FAO-UNESCO soil types [41], which were then further reclassified according to SWAT soil format. Finally, river discharge data for the Ca River, which are used for comparisons with modeled river discharge data in the calibration and validation processes, were available for Yen Thuong hydrological station. These river discharge data, collected on a daily basis, were mostly provided by the Hydro-Meteorological Data Center, except for data for certain years, which were provided by the Vietnam Institute of Meteorology, Hydrology and Environment.

### 2.4. Accuracy Assessment

Calibration and validation of the SWAT model were performed using observed river discharge data collected at Yen Thuong hydrological station. For convenience, the total available historical river discharge data (1971–2010) was divided into two sets: 25 years (1971–1995) for calibration and 15 years (1996–2010) for validation. The watershed characteristics, including land use, soil properties, and agricultural management factor, were kept constant throughout the whole simulation period. To evaluate the model predictions for both time periods, we used several different statistical indicators, including the coefficient of determination (*R* ^2^), Nash-Sutcliffe simulation efficiency (NSE), percent bias (PBIAS), and root mean square error-observation standard deviation ratio (RSR).

The *R* ^2^ value describes the degree of collinearity between simulated and observed data [42]. In other words, it is an indicator of the strength of the relationship between simulated and observed data [43]. Meanwhile, the NSE is a normalized statistic that determines the relative magnitude of the residual variance compared to the observed data variance [44]. NSE indicates how well the plot of observed versus simulated data fits the 1 : 1 line [43]. NSE is calculated as follows:

(1) $$\begin{array}{c} \text{NSE}=1-\frac{\sum_{i=1}^{n}{\left(Q_{i}^{\text{obs}}-Q_{i}^{\text{sim}}\right)}^{2}}{\sum_{i=1}^{n}{\left(Q_{i}^{\text{obs}}-Q^{\text{obsmean}}\right)}^{2}}, \end{array}$$

where *Q*_*i*_^obs^ and *Q*_*i*_^sim^ are the observed and simulated values of the river discharge, respectively; *Q* ^obsmean^ is the mean of the observed values of the river discharge; and *n* is the total number of observations. NSE varies from zero to one. Essentially, the closer the NSE is to 1, the more accurate the model is.

PBIAS is used to determine if the average tendency of the simulated data is larger or smaller than their observed counterparts [45]. PBIAS is computed as shown in the following equation, with all parameters sharing the same definitions shown in (1):

(2) $$\begin{array}{c} \text{PBIAS}=\frac{\sum_{i=1}^{n}\left(Q_{i}^{\text{obs}}-Q_{i}^{\text{sim}}\right)}{\sum_{i=1}^{n}Q_{i}^{\text{obs}}}*100\%. \end{array}$$

The ideal value of PBIAS is zero, with low-magnitude values indicating accurate model simulation. Positive values indicate model underestimation bias, and negative values indicate model overestimation bias [45].

RSR is a development of root mean square error (RMSE), which is one of the most frequently used error index statistics [46]. RSR standardizes RMSE using the observations standard deviation and is calculated as the ratio of the RMSE and the standard deviation of observed data, as displayed in

(3) $$\begin{array}{c} \text{RSR}=\frac{\text{RMSE}}{{\text{STDEV}}_{\text{obs}}}=\frac{\sqrt{\sum_{i=1}^{n}{\left(Q_{i}^{\text{obs}}-Q_{i}^{\text{sim}}\right)}^{2}}}{\sqrt{\sum_{i=1}^{n}{\left(Q_{i}^{\text{obs}}-Q^{\text{obsmean}}\right)}^{2}}}. \end{array}$$

All parameters in (3) share the same definitions as shown in (1).

RSR incorporates the benefits of error index statistics (i.e., RMSE) and includes a scaling/normalization factor (i.e., STDEV), so that the resulting statistic and reported values can apply to various constituents [42]. The ideal value of RSR is 0, which indicates zero RMSE or residual variation and therefore perfect model simulation. The lower the RSR, the lower the RMSE and the better the model simulation performance.

According to Moriasi et al. [42], river discharge prediction can be judged as satisfactory if NSE > 0.50 and RSR ≤ 0.70 and PBIAS ≤ ±25. Thus, all the values of NSE, PBIAS, and RSR were compared with those recommended by Moriasi et al. [42], which are displayed in Table 3.

## 3. Results and Discussion

### 3.1. Calibration and Validation of Hydrological Simulation

**Figure 2:**
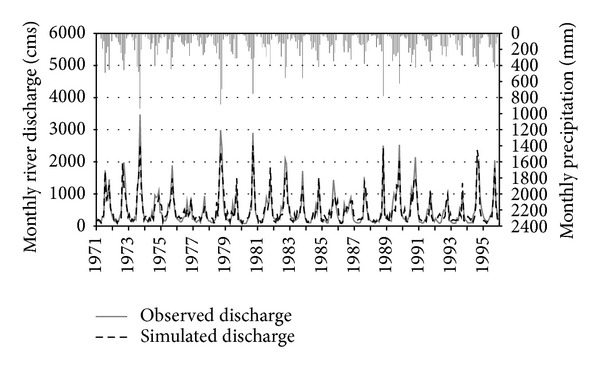
Monthly time series comparison of simulated versus observed river discharge at Yen Thuong Station with reference to monthly precipitation during the 25-year calibration period (1971–1995).

**Figure 3:**
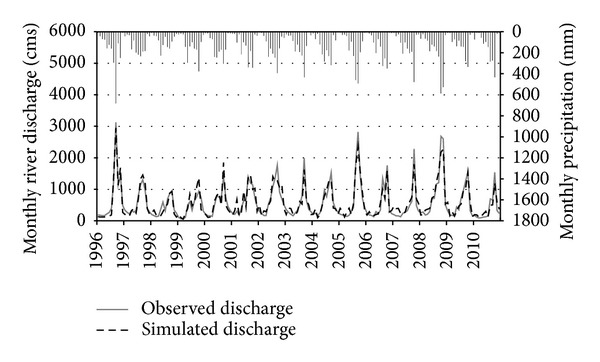
Monthly time series comparison of simulated versus observed river discharge at Yen Thuong Station with reference to monthly precipitation during the 15-year validation period (1996–2010).

**Figure 4:**
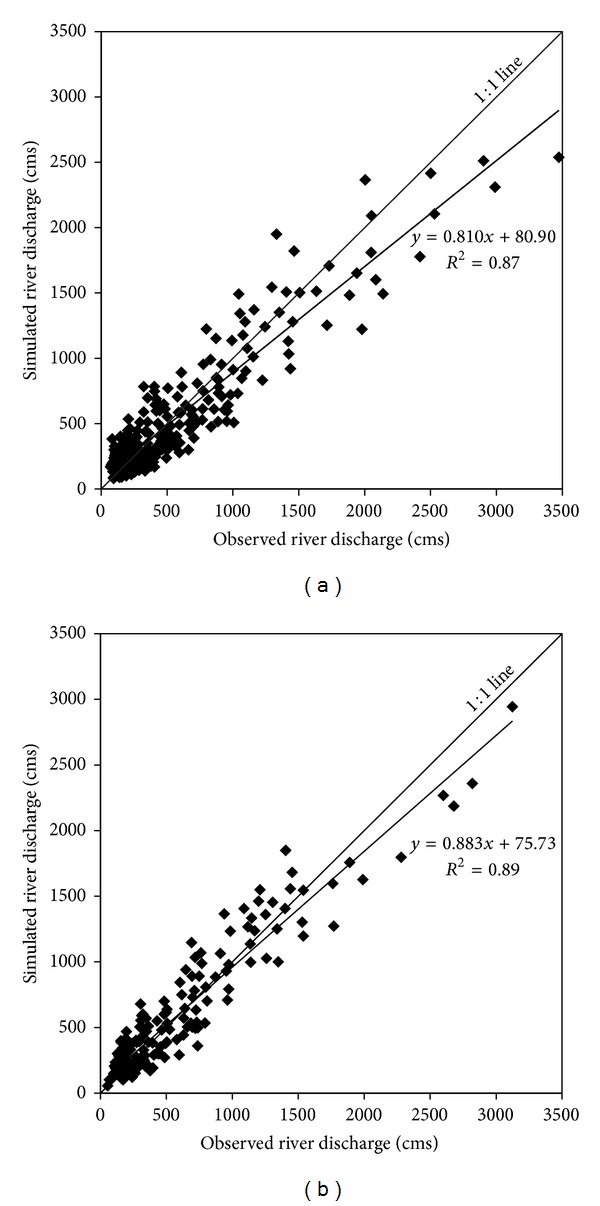
Regression plots of simulated versus observed discharge relative to 1 : 1 line for (a) 25-year calibration period (1971–1995) and (b) 15-year validation period (1996–2010).

Figures 2 and 3 graphically illustrate the time series comparison of simulated and observed cumulative monthly river discharge with reference to monthly precipitation for the Ca River at Yen Thuong Station, over the 25-year calibration (1971 through 1995) and 15-year validation (1996 through 2010), respectively. Overall, the SWAT model accurately tracked the observed river discharge for both time periods, although some of the low flow months were overpredicted and most of the peak flow months were underpredicted. Compared to the calibration period, in the validation period the simulated discharge followed more closely the corresponding observed discharge, with less underprediction of peak flow months and less overprediction of low flow months. This can also be seen in the regression plots (Figures 4(a) and 4(b)), where the linear trend line computed for the validation period was closer to the 1 : 1 line than in the calibration period. The regression plots also show that underprediction primarily occurred for the high discharge values, whilst overprediction mostly occurred for low discharge values. Evaluation statistics computed for both time periods are presented in Table 4. All of them showed a very good performance rating relative to the guidelines recommended by Moriasi et al. [42], which were previously shown in Table 3. Again, the performance of SWAT for the validation period was better than for calibration period as all of the statistics computed for this period were stronger than those computed for the calibration period. A positive bias of 3.17 percent was found for the calibration period, whereas the validation period resulted in a negative bias of −1.44 percent. This indicates that there were a model underestimation bias for the calibration period and an overestimation bias for the validation period. However, the magnitude of these biases was insignificant.

From the results of a study conducted in the Save catchment in southwest France, Oeurng et al. [47] concluded that a significant fluctuation in the hydrological regime may cause difficulties for discharge calibration. In contrast to that, it should be noted that the hydrological regime of the Ca River in our study was highly predictable, with low flows occurring in February, March, and April and peak flows occurring in August, September, and October. This appears to have led to an effective discharge calibration, which then resulted in accurate predictions. However, it should also be noted that, although in general the performance of the model for both time periods was assessed as “very good,” the ability of SWAT to predict flood discharge when the river has overflowed is not very high since it underestimated most of the peak flows. In some months, for example, September 1973, September 1978, and October 1990, the peak simulated discharges were approximately 1,000 cms (around 30%) smaller than the peak observed in the corresponding actual discharges. According to Luo et al. [48], one explanation for the problem of underestimation in SWAT is the assumption behind the model that water entering deep aquifers is not included in the water budget but is considered lost from the system. In addition, Beven [49] argued that the setting of model parameters to obtain the highest NSE value may cause underestimation due to the parameter equifinality or overparameterization problem. This usually leads to the issue of model parameter uncertainty and model complexity control for hydrological prediction [50].

### 3.2. Projected Temperature Changes

**Figure 5:**
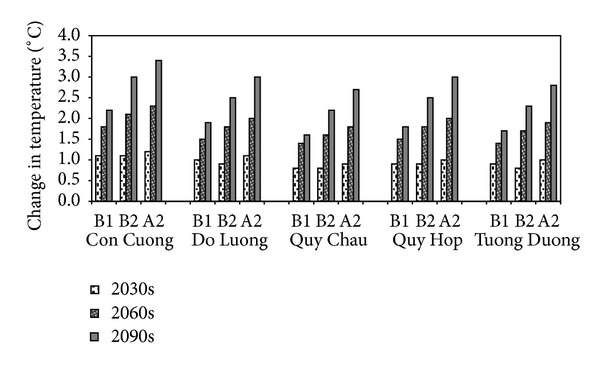
Relative change in mean annual temperature under 3 scenarios at 5 stations of the UCRW.

In all 3 scenarios for the 5 stations investigated, temperature increases gradually throughout the 21st century, although the degree of increment is rather different among stations. At Con Cuong station, the temperature rises at the highest rate (up to 3.4°C in the 2090s under scenario A2), followed by Do Luong and then Quy Hop stations. Temperature increases least at Quy Chau station (Figure 5). On average, according to the high emission scenario (A2), in the UCRW, increases of 1.0°C, 2.0°C, and 3.0°C are expected for the periods of the 2030s, 2060s, and 2090s, respectively. It should be noted that the behavior of scenarios B1, B2, and A2 is fairly similar for the mean annual temperature until the near future period (2030s), with an increase of approximately 1°C compared to the baseline period. The difference in behavior increases slightly in the middle future period (2060s). From then on, the A2 scenario simulation predicts the largest changes, followed by the B2 and then the B1 scenario. In the 2090s, the differences between scenario A2 and scenarios B2 and B1 are approximately 0.5°C and 1.2°C, respectively. This is consistent with the characteristics of the emission scenarios, which evolve similarly until the middle of the 21st century, when A2 becomes more negative due to the continuous increase of population growth and, therefore, the increase in greenhouse gas emissions. In contrast, B1 becomes less negative due to the slowing of population growth, with a corresponding reduction in greenhouse gas emissions [8, 51]. The same behavior has also been reported in several very recent studies using IPCC AR3 or IPCC AR4 models on a regional scale [52–54].

### 3.3. Impact of Climate Change on PET

**Figure 6:**
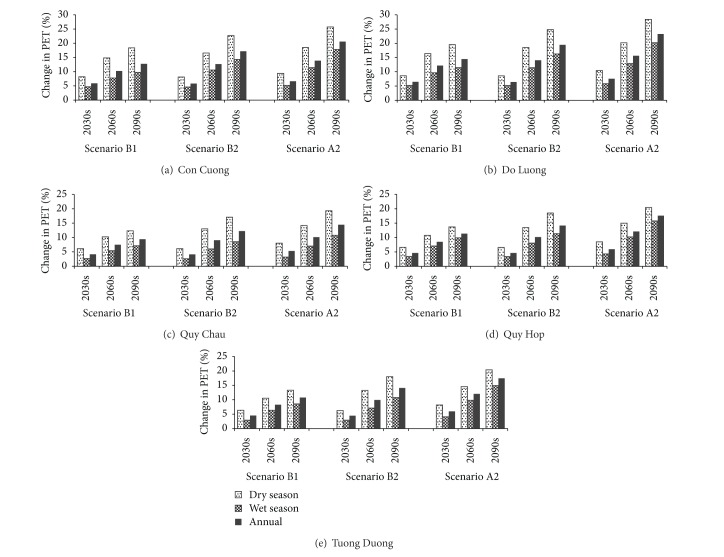
Relative change of PET under three emission scenarios at five stations of the UCRW.

PET of future periods is calculated by the Hargreaves equation, using downscaled temperature data [40, 55].  Figure 6 graphically illustrates the relative changes in seasonal and annual PET at 5 meteorological stations of the UCRW under 3 emission scenarios. The patterns of changes in the PET at the 5 investigated stations are very similar, although the magnitude of the changes is different among the stations. In general, PET increases gradually in both the wet and dry seasons throughout three time-stages, resulting in a continuous increase of annual PET. The increase rate in the dry season is around half as much again as that of the wet season. The largest increase rate was observed at Con Cuong station, followed by Do Luong and then Quy Hop. PET increases least at Tuong Duong station. This order is consistent with that of the temperature scenarios previously shown in Figure 5.

In addition, similar to those for temperature, the behaviors of the three scenarios for PET are almost the same for the near future period (2030s) and become increasingly different by the middle future period (2060s) and significantly different by the end of this century, with the largest increase occurring with the A2 scenario, followed by the B2 and then the B1 scenario.

Averaging the five investigated stations, according to scenario A2, by end of this century, a 23% increase in the dry season, a 16 % increase in the wet season, and a 19% increase in the annual PET are expected. The corresponding rates for scenario B2 are 20%, 12%, and 15%, whereas for scenario B1 they are approximately 15%, 9%, and 12%, respectively.

It must be noted that the uncertainty in the predicted future seasonal mean and annual mean PET is large, and the increasing trends become more evident throughout the three different future stages. Such increasing trends are primarily driven by the increases in future predicted temperature, which is the main factor affecting the future PET in the SWAT model. This is similar to the finding of Xu et al. [56].

### 3.4. Impact of Climate Change on Precipitation

Our projection shows that precipitation in the UCRW is likely to change, varying by months and by observation stations. Precipitation is predicted to increase for February and all months from July to December and to decrease for January, March, April, and May. This pattern applies to all of the five investigated stations and throughout the 3 future time periods. For June, the pattern of change is not consistent among the 5 stations. Thus, at Do Luong, Quy Chau, and Quy Hop stations, precipitation is projected to increase, whereas, at Con Cuong and Tuong Duong stations, it is projected to decrease. However, it should be noted that the magnitude of the decrease in precipitation at these two stations is small, at around 1%, depending on the scenario.

**Figure 7:**
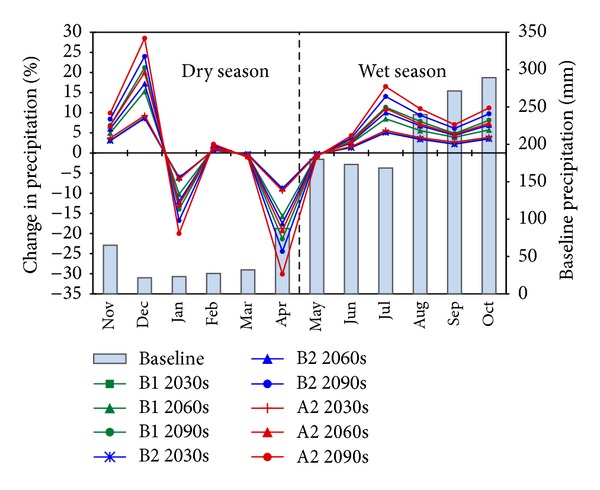
Change in monthly mean precipitation (line chart) relative to average data of the baseline period (column chart).

Figure 7 shows the relative change in monthly mean precipitation of the UCRW, computed as the average of the five investigated meteorological stations. It can be seen that there will likely be significant decreases in precipitation in January and April, with rates of up to 20% for January and 30% for April in the 2090s according to the high emission scenario A2. On the opposite side of the graph, the largest increase in precipitation is likely to occur in December, with the highest rate up almost 29% in the 2090s in scenario A2, followed by July and then October. Precipitation change in March and May is expected to be at a very small rate. Note that the months around October are in the flooding season, with a monthly precipitation of about 300 mm. A great increase in precipitation around this month is likely to cause a high risk of flooding to the UCRW.

Considering the 3 scenarios, the trends of precipitation change in each month projected by the 3 scenarios are clear, and, within each specific scenario, these trends are consistent throughout the century. In addition, the 3 scenarios behave similarly until the period of the 2030s, as the 3 lines B1 2030, B2 2030, and A2 2030 almost coincide. The order of the 3 scenarios with respect to the magnitude of precipitation change until the 2030s is A2 > B1 > B2. From the 2030s on, this order shifts to A2 > B2 > B1, with the difference among the scenarios growing larger throughout the century.

**Figure 8:**
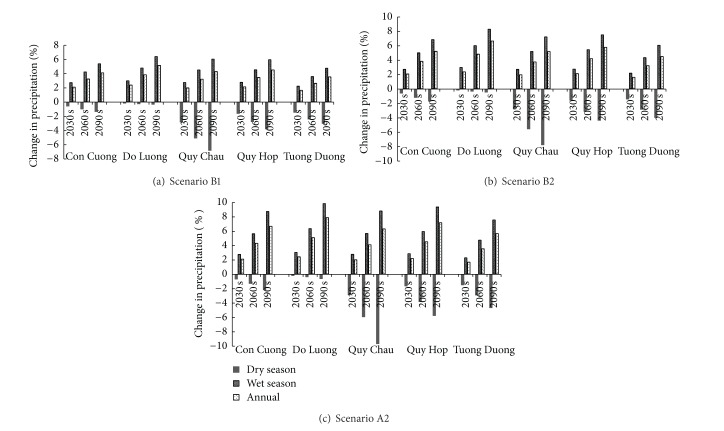
Relative change in mean seasonal and mean annual precipitation of five stations for three future periods under three different scenarios.

As stated earlier, climate in the UCRW can be divided into two seasons: the dry season, lasting from November to April, and the wet season, lasting from May to December. The amount of precipitation in the wet season accounts for more than 80% of the total annual precipitation. In this study, we investigated the seasonal change in precipitation, which is one of the most important factors resulting in drought or flooding problems in the watershed studied.  Figure 8 represents the relative change in mean seasonal and mean annual precipitation of five stations under three different scenarios.

Although the magnitude of the changes varies depending on the station and scenario, the overall pattern of the changes is obvious: there will likely be more precipitation in the wet season and less precipitation in the dry season. The degree of this change increases gradually throughout the three future periods. The greatest decrease in precipitation in the dry season is predicted to occur at Quy Chau station (down 9.7% by the 2090s according to scenario A2). On the other hand, the greatest increase in precipitation in the wet season is predicted to occur at Do Luong station (9.9% by the 2090s according to scenario A2). However, the rate of precipitation decrease at Do Luong station is predicted to be very small, at around 0.5%.

In general, at most of the investigated stations, the rate of precipitation increase in the wet season is greater than that of the precipitation decrease in the dry season (except for Quy Chau station, the rates of which are almost equal). In addition, as the amount of precipitation in the wet season is much greater than that in the dry season (80% compared to 20% of the annual precipitation), annual precipitation increases. The largest increase in annual precipitation is likely to occur at Do Luong station (7.9% in the 2090s according to scenario A2), followed by Quy Hop (7.2%), Con Cuong (6.7%), Quy Chau (6.4%), and Tuong Duong (5.7%).

In short, the uncertainty in the projected future precipitation in the UCRW is large. Large increases are likely to occur in the wet season, while large decreases are likely to occur in the dry season. Furthermore, although these two factors taken together lead to the prediction that annual precipitation will increase, there is likely to be a higher risk of drought in the dry season and a higher risk of flooding in the wet season.

### 3.5. Impact of Climate Change on River Discharge

**Figure 9:**
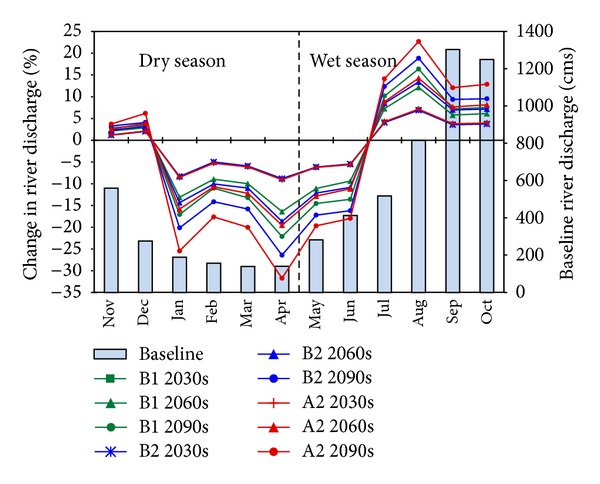
Monthly change in river discharge at Yen Thuong Station (line chart) relative to the average data of the baseline period (column chart).

A change of river discharge is projected for Yen Thuong hydrological station, which drains an area of approximately 22,800 km^2^.  Figure 9 illustrates the relative monthly change projected by the three climate change scenarios B1, B2, and A2 and displayed for the three future time periods of the 2030s, 2060s, and 2090s. There is a clear trend in changes throughout the year. Discharge is projected to decrease for the first six months of the year, from January to June, and increase for the other six months, from July to December. The magnitude of changes varies depending on the month and scenario. Note, however, that November and December belong to the dry season, whereas May and June belong to the wet season. Therefore, in the dry season, there are four months with decreases and two months with increases in discharge. On the other hand, discharge increases for four of the six months of the wet season and decreases for the other two months.

The largest increase in monthly discharge can be observed in August (7.1% in the 2030s, 14.3% in the 2060s, and 22.7% in the 2090s according to scenario A2), followed by July, October, and September. The smallest increase is likely to occur in November (1.2% in the 2030s according to scenario B2, 2.1% in the 2060s, and 2.8% in the 2090s according to scenario B1). The increase in discharge for all months from July to December can be explained by the large increases in precipitation for these months (Figure 7). However, the shapes of the increases in discharge (Figure 9) and precipitation (Figure 7) are not homogeneous. This is because of differences in temperature change and therefore differences in the rate of evapotranspiration change, in addition to the differences in the amount of precipitation between the months. For instance, precipitation increases most in December (in terms of percentage), but discharge in December increases with a small rate. This is due to the increase in PET in this month and also to the fact that the amount of precipitation in December is very small (only around 20 mm). Therefore, even a great increase in precipitation in this month could not produce a large change in discharge.

On the other side of the graph, the most substantial decrease can be found in April (9.1% in the 2030s, 19.6% in the 2060s, and 31.8% in the 2090s, according to scenario A2), followed by January, March, and May. The decrease in discharge in January, March, April, and May corresponds to the decrease in precipitation in these months (Figure 7). It is noticeable that discharge decreases in February and June, despite the increase in precipitation in these months. In February, the increase of about 1% of a small precipitation amount (about 20 mm) cannot compensate for the increase of PET driven by the temperature rise, resulting in a discharge decrease. June is one of the hottest months in the UCRW, with an average maximum temperature of about 38°C and an average PET of more than 100 mm. The increase in PET in this month is possibly more significant than the increase in precipitation (Figure 7); the river discharge therefore decreases. The differences in the trends in precipitation change and discharge change in June may also be due to the time-lag between the precipitation events and the stream discharges. In addition to evaporation, saturation is also an important factor. When it rains, it takes time for the ground to become saturated, but once it has become saturated, any additional rainfall then runs over the land into streams. However, the increase in temperature in the month of June can cause an increase in the ground infiltration rate, resulting in a decrease in the amount of water running into streams.

**Figure 10:**
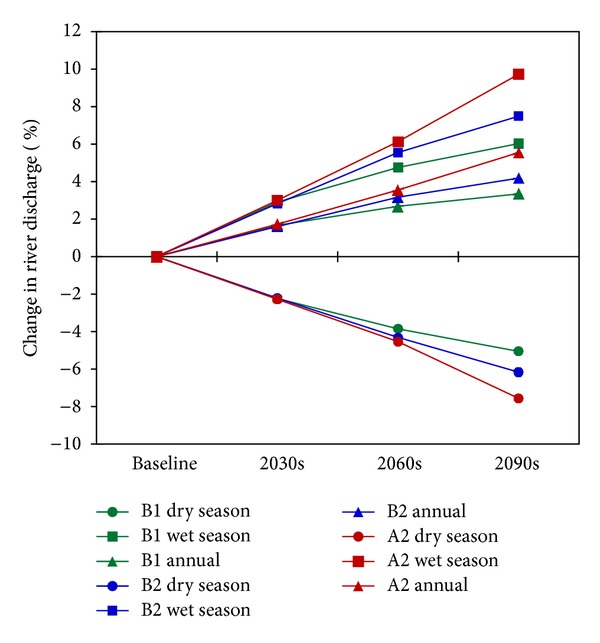
Change in seasonal and annual river discharge at Yen Thuong Station.

Figure 10 represents projected future seasonal and annual changes in river discharge, computed from monthly changes. Overall, it can be seen that dry season discharge is projected to decrease, while wet season discharge and annual discharge are likely to increase. This trend is obvious for all three scenarios. There is a similarity among the three scenarios until the period of the 2030s, with a prediction for dry season discharge to decrease by around 2.2% and wet season discharge to increase by approximately 3.0%. From the 2030s on, the magnitude of change between the three scenarios increases. Discharge is likely to change the most quickly under scenario A2, faster than under scenario B2 and faster still than under scenario B1. This pattern is consistent with the patterns of changes in temperature (Figure 5), PET (Figure 6), and precipitation (Figure 8).

Specifically, increases of 4.8%, 5.6%, and 6.1% in wet season discharge are expected for the 2060s, under scenarios B1, B2, and A2, respectively. The corresponding rates for the 2090s are 6.0%, 7.5%, and 9.7%. For dry season discharge, in the 2060s, decreases of 3.9% (B1), 4.3% (B2), and 4.5% (A2) are likely to occur. In the 2090s, larger decreases can be expected: 5.1% according to scenario B1, 6.2% according to scenario B2, and 7.6% according to scenario A2. Note that wet season discharge is much higher than dry season discharge (763 m^3^/s compared to 243 m^3^/s, computed as the average of the 20-year baseline period 1980–1999), and the increase in wet season discharge is more significant than the decrease in dry season discharge, so that annual discharge increases. Under scenario A2, the increase rate in annual discharge may be up to 3.6% in the 2060s and 5.6% in the 2090s.

Overall, annual discharge is projected to increase, meaning that there will be more water in the watershed annually, but there is also likely to be a problem with uneven distribution of water resources: a large volume of water in the wet season and a scarcity of water in the dry season. This indicates the possibility of more frequent floods in the wet season and droughts in the dry season.

## 4. Conclusion

In this study, the potential impacts of climate change on water resources in the UCRW were assessed using downscaled GCMs and the SWAT model. We focused on the seasonal trends of PET, precipitation, and especially river discharge. Climate change projections under three emission scenarios, B1, B2, and A2, presented similar results until the near future period (2030s). From then on, scenario A2 resulted in the largest changes (for both increase and decrease in climatic factors, that is, temperature, precipitation, and PET), followed by scenario B2 and then B1. The differences between scenarios become larger with time. While there were substantial differences between the scenarios, general trends can be drawn from them. All of the simulations indicate the following, in varying magnitudes: temperature rises, increased PET in both wet and dry seasons, increased precipitation in the wet season, and decreased precipitation in the dry season. Consequently, hydrological simulation by SWAT resulted in similar predictions for three scenarios until the 2030s, but diverging from then on. The simulation indicates likely increases in wet season discharge and decreases in dry season discharge. The magnitudes of these changes increase with time. Overall, climate change is likely to exacerbate the already seasonally uneven distribution of water resources, implying more severe and frequent droughts and floods in the UCRW. The results of this study should therefore be useful for the effective management of water resources in the watershed, especially for flooding and drought effect prevention initiatives.

## Figures and Tables

**Table 1:** Global climate models used for climate change scenarios generation in this study.

CMIP3 designator	Origin	First publication	Name in SCENGEN	Resolution (deg.)
BCCR-BCM2.0	Norway	2005	BCCRBCM2	2.8 × 2.8
CCSM3	USA	2004	CCSM—30	1.4 × 1.4
CGCM3.1(T47)	Canada	2005	CCCMA-31	3.75 × 3.75
CNRM-CM3	France	2004	CNRM-CM3	2.8 × 2.8
CSIRO-Mk3.0	Australia	2001	CSIRO-30	1.875 × 1.875
ECHAM5/MPI-OM	Germany	2005	MPIECH-5	2.0 × 2.0
ECHO-G	Germany/Korea	2001	ECHO—G	1.4 × 1.4
FGOALS-g1.0	China	2004	FGOALS1G	2.8 × 3.0
GFDL-CM2.0	USA	2005	GFDLCM20	2.5 × 2.0
GFDL-CM2.1	USA	2005	GFDLCM21	2.5 × 2.0
GISS-EH	USA	2004	GISS—EH	5.0 × 4.0
GISS-ER	USA	2004	GISS—ER	5.0 × 4.0
INM-CM3.0	Russia	2004	INMCM-30	5.0 × 4.0
IPSL-CM4	France	2005	IPSL_CM4	3.75 × 2.5
MIROC3.2(hires)	Japan	2004	MIROC-HI	1.1 × 1.1
MIROC3.2(medres)	Japan	2004	MIROCMED	2.8 × 2.8
MRI-CGCM2.3.2	Japan	2003	MRI-232A	2.8 × 2.8
PCM	USA	1998	NCARPCM1	2.8 × 2.8
UKMO-HadCM3	UK	2000	UKHADCM3	3.75 × 2.5
UKMO-HadGEM1	UK	2006	UKHADGEM	1.875 × 1.25

**Table 2:** Meteorological stations investigated in this study.

Station name	Longitude	Latitude	Data available	Mean annual precipitation (mm)	Mean annual temperature (°C)	Mean annual PET (mm)	Mean annual humidity (%)
Con Cuong	104°54′E	19°02′N	1971–2010	1696	23.8	830	85.4
Do Luong	105°18′E	18°54′N	1971–2010	1822	23.9	855	84.4
Quy Chau	105°07′E	19°34′N	1971–2010	1663	23.4	745	85.8
Quy Hop	105°09′E	19°19′N	1971–2010	1603	23.4	883	83.5
Tuong Duong	104°26′E	19°17′N	1971–2010	1254	24.0	887	81.4

**Table 3:** Performance rating of evaluation statistic for monthly river discharge.

Performance rating	NSE	PBIAS (%)	RSR
Very good	0.75 < NSE ≤ 1.00	PBIAS < ±10	0.00 ≤ RSR ≤ 0.50
Good	0.65 < NSE ≤ 0.75	±10 ≤ PBIAS ≤ ±15	0.50 < RSR ≤ 0.60
Satisfactory	0.50 < NSE ≤ 0.65	±15 ≤ PBIAS ≤ ±25	0.60 < RSR ≤ 0.70
Unsatisfactory	NSE ≤ 0.50	PBIAS ≥ ±25	RSR > 0.70

**Table 4:** Evaluation statistics of hydrological simulation.

Statistics	Calibration	Validation
NSE	0.86	0.89
*R* ^2^	0.87	0.89
PBIAS (%)	3.17	−1.44
RSR	0.37	0.32
